# Sympathoadrenergic modulation of hematopoiesis: a review of available evidence and of therapeutic perspectives

**DOI:** 10.3389/fncel.2015.00302

**Published:** 2015-08-05

**Authors:** Marco Cosentino, Franca Marino, Georges J. M. Maestroni

**Affiliations:** Center for Research in Medical Pharmacology, University of InsubriaVarese, Italy

**Keywords:** dopamine, noradrenaline, adrenaline, adrenoceptors, dopaminergic receptors, hematopoiesis, neuroimmune phamacology, drug repurposing

## Abstract

Innervation of the bone marrow (BM) has been described more than one century ago, however the first *in vivo* evidence that sympathoadrenergic fibers have a role in hematopoiesis dates back to less than 25 years ago. Evidence has since increased showing that adrenergic nerves in the BM release noradrenaline and possibly also dopamine, which act on adrenoceptors and dopaminergic receptors (DR) expressed on hematopoietic cells and affect cell survival, proliferation, migration and engraftment ability. Remarkably, dysregulation of adrenergic fibers to the BM is associated with hematopoietic disturbances and myeloproliferative disease. Several adrenergic and dopaminergic agents are already in clinical use for non-hematological indications and with a usually favorable risk-benefit profile, and are therefore potential candidates for non-conventional modulation of hematopoiesis.

## Introduction

**Figure 1:**
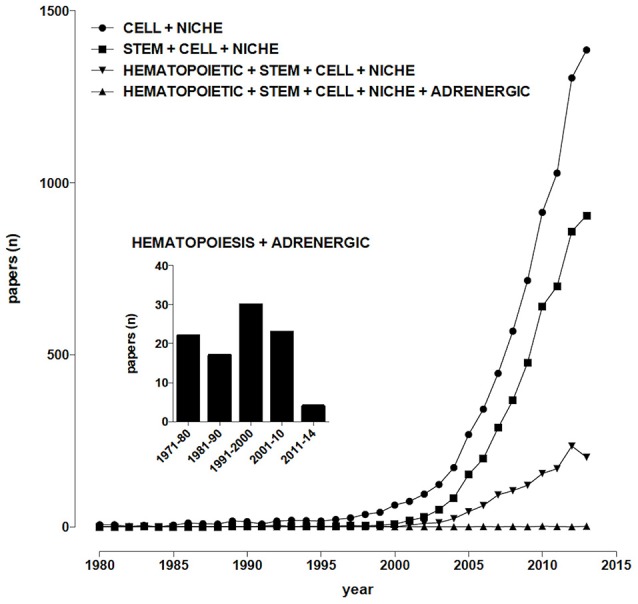
**Temporal trends of papers indexed in PubMed**. (Alexandru Dan Corlan. Medline trend: automated yearly statistics of PubMed results for any query, 2004. Web resource at URL: http://dan.corlan.net/medline-trend.html. Accessed: 2015-03-24. Archived by WebCite at http://www.webcitation.org/65RkD48SV).

The term “niche”, derived from the Latin word “mytilus” (mussel), has eventually come to designate a shallow recess in a wall, as for a statue or other decorative object, in view of the similarity with the shape of a seashell, and broadly a place suitable or appropriate for a person or thing. In biology and medicine, the use of “niche” to designate the microenvironment where cells are found, and which may determine their fate, becomes increasingly popular in the early 90’s of the last century, thereafter steadily rising, from 27 papers/year on average in the period 1991–2000 (including about 3, 4 dealing with stem cells) to more than 1000/year since 2011 (about two thirds of them dealing with stem cells; Figure 1). So far, niches for several types of stem cells have been identified and characterized, including neurogenic (Bjornsson et al., 2015), osteogenic (Bianco, 2011), epithelial (Secker and Daniels, 2009), hematopoietic (Mendelson and Frenette, 2014).

The hematopoietic stem cell (HSC) niche as an organized microenvironment that controls HSC homeostasis was first proposed in Schofield (1978) and thereafter much progress has been made in characterizing the different cell types that are essential in HSC maintenance and regeneration (Lymperi et al., 2010; Wang and Wagers, 2011; Mendelson and Frenette, 2014), including perivascular stromal cells, reticular cells, endothelial cells, macrophages as well as sympathoadrenergic nerve terminals.

Sympathetic fibers innervating the bone marrow (BM) were described at least 70 years ago (Kuntz and Richins, 1945), their stimulation resulting in the release of reticulocytes and neutrophils into systemic circulation (DePace and Webber, 1975), however for many years their role was mainly related to the regulation of the permeability of the venous sinusoids and the mobility of BM cells, until the evidence was provided that chemical sympathectomy increases the number of peripheral blood leukocytes after syngeneic BM transplantation in mice, an effect which is mimicked by the α_1_-adrenoceptor antagonist prazosin (Maestroni et al., 1992). Nowadays, sympathetic nerves are considered, together with the hypothalamus-pituitary-adrenal axis, the main communication pathway between the brain and the immune system (Elenkov et al., 2000; Marino and Cosentino, 2013) and about one hundred papers have been published dealing with adrenergic modulation of hematopoiesis (Figure 1). It appears therefore that, despite sympathetic innervation of the BM has been known for decades, sympathoadrenergic modulation of hematopoiesis involves so far relatively few scientists around the world, a somewhat paradoxical observation in view of the many significant therapeutic opportunities which could arise from this field of research.

We will hereafter review current knowledge on innervation of the BM and on sympathoadrenergic modulation of hematopoiesis, discussing available evidence in light of the opportunity to repurpose adrenergic (and possibly also dopaminergic) agents as modulators of hematopoiesis. Indeed, any dirrectly and indirectly acting adrenergic and dopaminergic therapeutics are currently used for non-hematological indications, and could thus represent an attractive source of non-conventional agents for the modulation of the hematopoietic process. To this end, a brief general introduction to the neuroimmune pharmacology of catecholamine neurotransmitters will be first provided.

## Neuroimmune Pharmacology of Catecholamine Neurotransmitters

**Figure 2:**
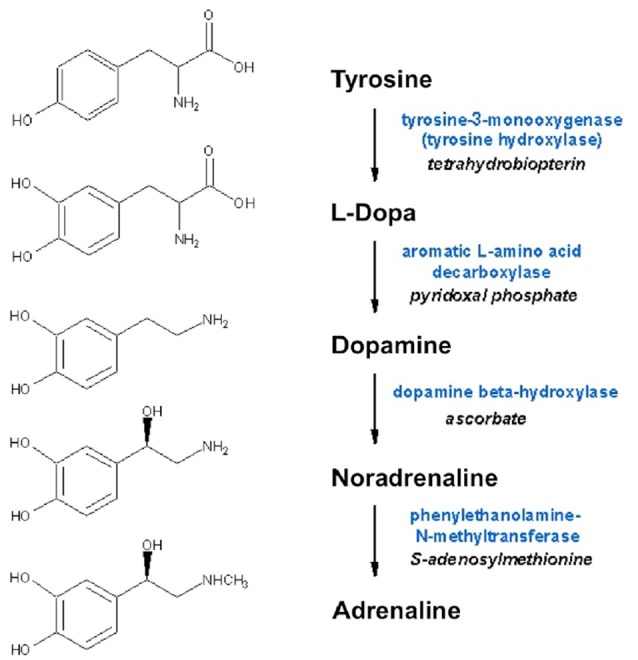
**Biosynthesis of dopamine, noradrenaline and adrenaline**. Synthesizing enzymes and enzyme cofactors are shown close to each arrow.

Noradrenaline is a neurotransmitters in the central and peripheral nervous systems, and to a lesser extent a neurohormone in chromaffin cells in medulla of adrenal glands. From the *locus coeruleus* (LC), axons project rostrally, dorsally, and caudally to spinal cord, affecting attention, arousal and vigilance, and regulating hunger and feeding behavior. Adrenaline is a minor neurotransmitter in the central nervous system (CNS), however it is the main neurohormone secreted by the adrenal medulla. In periphery, noradrenaline is the main transmitter of sympathetic postganglionic fibers. Peripheral adrenergic actions include: smooth muscles contraction (skin, kidney, and mucous membranes blood vessels), stimulation of sweat glands, relaxation gut wall, bronchi, skeletal muscle blood vessels, increases of heart rate and contraction force. In addition, they have prominent metabolic (increased liver and muscle glycogenolysis, increased lipolysis) and endocrine actions (e.g., modulation of insulin and renin secretion). Dopamine is a key neurotransmitter in the brain, where it is involved in a wide variety of CNS functions including motivation, cognition, movement and reward. Besides being biochemically and metabolically related (since are all produced from the non-essential amino acid tyrosine; Figure 2), several lines of evidence suggest that dopamine may be stored in and released from sympathetic nerve terminals, thus acting as a transmitter even at this level (Bell, 1988; Bencsics et al., 1997). Detailed discussion of dopamine, noradrenaline and adrenaline neurochemistry, anatomy and physiology can be found in Feldman et al. (1997).

### Pharmacology of Dopamine, Noradrenaline and Adrenaline

Dopamine, noradrenaline and adrenaline act on 7-transmembrane, G-protein coupled receptors. Dopaminergic receptors (DR) exist in five different molecular subtypes, grouped into two families according to their pharmacology and second messenger coupling: the D_1_-like (D_1_ and D_5_) activating adenylate cyclase and the D_2_-like (D_2_, D_3_ and D_4_) inhibiting adenylate cyclase (Beaulieu and Gainetdinov, 2011; Alexander et al., 2013; Cosentino et al., 2013). Adrenoceptors (ARs) are nine different receptors, including three major types—α_1_, α_2_ and β—each further divided into three subtypes (Alexander et al., 2013). DR agonists are used to treat Parkinson’s disease (PD), restless leg syndrome, and hyperprolactinemia, while antagonists are used as antipsychotics and antiemetics (Table 1). AR agonists and antagonists are used to treat hypertension, angina pectoris, congestive heart failure, asthma, depression, benign prostatic hypertrophy, and glaucoma, as well as other conditions such as shock, premature labor and opioid withdrawal, and as adjunct medications in general anaesthesia (Table 2). Pharmacological modulation of adrenergic and dopaminergic pathways can be obtained also by use of indirectly acting agents. All the steps involved in dopamine, noradrenaline and adrenaline synthesis, storage and release, uptake and metabolism represent the target of several drugs already in use for non-immune indications (e.g., cardiovascular, neurologic, neuropsychiatric). Pharmacological targets and examples of therapeutic drugs are listed in Tables 3 and 4 (Cosentino et al., 2013).

**Table 1:** **Examples of dopaminergic agonists and antagonists currently used as therapeutic drugs (brand names in parentheses)**.

**Agonists**		
**D_1_-like**	Fenoldopam mesylate (Corlopam)
**D_1_-like/D_2_-like**	Ergot Alkaloids: bromocriptine (Parlodel); pergolide (Permax); cabergoline (Dostinex) Non-Ergot Alkaloids: apomorphine (Apokyn); rotigotine (Neupro)
**D_2_-like**	Non-Ergot Alkaloids: pramipexole (Mirapex); ropinirole (Requip)
**Antagonists**	
	Typical antipsychotics chlorpromazine (Thorazine), fluphenazine (Prolixin), haloperidol (Haldol), loxapine (Loxitane), molindone (Moban), perphenazine (Trilafon), pimozide (Orap), thioridazine (Mellaril), thiothixene (Navane), trifluoperazine (Stelazine)
	A typical antipsychotics amisulpride (Solian), clozapine (Clozaril), olanzapine (Zyprexa), quetiapine (Seroquel), risperidone (Risperdal), sulpiride (Dogmatil), ziprasidone (Geodon)
	Antiemetics domperidone, metoclopramide (Reglan), prochlorperazine (Compazine)

**Table 2:** **Examples of dopaminergic agonists and antagonists currently used as therapeutic drugs**.

**α_1_AR**	
**Agonists**	Methoxamine, methylnorepinephrine, midodrine, oxymetazoline, metaraminol, phenylephrine
*Indications*	*Vasoconstriction and mydriasis, used as vasopressors, nasal decongestants and eye exams*
**Antagonists**	Alfuzosin, doxazosin, phenoxybenzamine, phentolamine, prazosin, tamsulosin, terazosin, trazodone
*Indications*	*Hypertension, benign prostatic hyperplasia*
**α_2_-AR**	
**Agonists**	Dexmedetomidine, medetomidine, romifidine, clonidine, brimonidine, detomidine, lofexidine, xylazine, tizanidine, guanfacine, amitraz
*Indications*	*Antihypertensives, sedatives and treatment of opiate dependence and alcohol withdrawal symptoms*
**Antagonists**	Phentolamine, yohimbine, idazoxan, atipamezole, trazodone, mianserin, mirtazapine
*Indications*	*Aphrodisiac, antidepressants, reversal of α_2_-AR agonist-induced sedation*
**β-AR**	
**β_1_-AR**	
**Agonists**	Dobutamine, isoprenaline, noradrenaline
*Indications*	*Bradycardia, heart failure, cardiogenic shock*
**Antagonists**	Metoprolol, atenolol, bisoprolol, propranolol, timolol, nebivolol
*Indications*	*Cardiac arrhythmia, congestive heart failure, glaucoma, myocardial infarction, migraine prophylaxis*
**β_2_-AR**		
**Agonists**	Short-acting: salbutamol, levosalbutamol, terbutaline, pirbuterol, procaterol, clenbuterol, metaproterenol, fenoterol, bitolterol mesylate, ritodrine, isoprenaline. Long-acting: salmeterol, formoterol, bambuterol, clenbuterolUltra-long-acting: indacaterol
*Indications*	*Asthma (effects: dilation of bronchial passages, vasodilation in muscle and liver, relaxation of uterine muscle, and release of insulin)*
**Antagonists**	Butoxamine, timolol, propranolol
*Indications*	*Glaucoma, heart attacks, hypertension, migraine headache; investigational: stage fright, PTSD*
**β_3_-AR**		
**Agonists**	Amibegron (*investigational*: *antidepressant, anxiolytic*), solabegron (*overactive bladder, irritable bowel syndrome*)
**Antagonists**	SR 59230A

**Table 3:** **Pharmacological targets for the modulation of dopaminergic and adrenergic pathways by agents targeting storage and release (brand/street names in parentheses)**.

**Reuptake inhibitors/transporter blockers**	
**DAT inhibitors**	Methylphenidate (Ritalin, Focalin, Concerta), bupropion (Wellbutrin, Zyban), amineptine (Survector, Maneon, Directin), nomifensine (Merital, Alival), cocaine, methylenedioxypyrovalerone (MDPV; “Sonic”), ketamine (K; Ketalar, Ketanest, Ketaset; “Special-K”, “Kit Kat”, etc.), phencyclidine (PCP; Sernyl; “Angel Dust”, “Rocket Fuel”, etc.)
*Indications*	*Attention-deficit hyperactivity disorder (ADHD), narcolepsy, obesity as anorectics, depression and anxiety, drug addiction, sexual dysfunction, illicit street drugs*
**VMAT2 inhibitors**	Reserpine (Serpasil), tetrabenazine (Nitoman, Xenazine), deserpidine (Harmonyl)
*Indications*	*Sympatholytics or antihypertensives, antipsychotics*
**Releasing agents**	Amphetamine (Adderall, Dexedrine; “Speed”), lisdexamfetamine (Vyvanse), methamphetamine (Desoxyn; “Meth”, “Crank”, “Crystal”, etc.), methylenedioxymethamphetamine (MDMA; “Ecstasy”, “E”, “X”, “XTC”, etc.), phenmetrazine (Preludin; “Prellies”), pemoline (Cylert), 4-methylaminorex (4-MAR; “Ice”, “Euphoria”, etc.), benzylpiperazine (BZP; “Bennies”, “A2”, “Sunrise”, “Frenzy”, etc.)
*Indications*	*Attention-deficit hyperactivity disorder (ADHD), narcolepsy, obesity, depression and anxiety, drug addiction, sexual dysfunction, illicit street drugs*
**“Activity enhancers”**	Benzofuranylpropylaminopentane (BPAP), phenylpropylaminopentane (PPAP)
*Indications*	*Investigational*: *Alzheimer’s disease, Parkinson’s disease and clinical depression*

*DAT, dopamine transporter; VMAT2, vesicular monoamine transporter type 2*.

**Table 4:** **Pharmacological targets for the modulation of dopaminergic and adrenergic pathways by agents targeting metabolism (brand/street names in parentheses)**.

**Reuptake inhibitors/transporter blockers**	
**Monoamine oxidase inhibitors**	Nonselective agents: phenelzine (Nardil), tranylcypromine (Parnate), isocarboxazid (Marplan)MAOA selective agents: moclobemide (Aurorix, Manerix) MAOB selective agents: selegiline (Eldepryl, Zelapar, Emsam), rasagiline (Azilect), pargyline (Eutonyl) Harmala alkaloids: harmine, harmaline, tetrahydroharmine, harmalol, harman, norharman *found to varying degrees in Nicotiana tabacum (Tobacco; also cigarettes, cigars, chew, hookah, etc.), Banisteriopsis caapi (Ayahausca, Caapi, Yage), Peganum harmala (Harmal, Syrian Rue), Passiflora incarnata (Passion Flower), and Tribulus terrestris (Puncture Vine), among others*
*Indications*	*Depression and anxiety, Parkinson’s disease (PD) and dementia, for the recreational purpose*
**Catechol O-methyl transferase (COMT) inhibitors**	Entacapone (Comtan, Stalevo), tolcapone (Tasmar), nitecapone
*Indications*	*Parkinson’s disease (PD)*
**DOPA decarboxylase (DDC) inhibitors**	Benserazide (Prolopa, Madopar, etc.), carbidopa (Lodosyn, Atamet, Parcopa, Sinemet, Stalevo, etc.), methyldopa (Aldomet, Aldoril, Dopamet, Dopegyt, etc.)
*Indications*	*Parkinson’s disease (PD), sympatholytic or antihypertensive agents*
**Dopamine β-hydroxylase (DBH) inhibitors**	Disulfiram (Antabuse)
*Indications*	*Drug addiction as an anticraving agent*
**Dopamine β-hydroxylase (DBH) inhibitors**	Disulfiram (Antabuse)
*Indications*	*Drug addiction as an anticraving agent*
**Tyrosine hydroxylase (TH) inhibitors**	Metirosine (Demser)
*Indications*	*Pheochromocytoma (PCC) as sympatholytic/antihypertensive agent*
**Others**	Hyperforin and adhyperforin [Hypericum perforatum (St. John’s Wort (SJW))], L-theanine [Camellia sinensis (Tea Plant, also known as Black, White, Oolong, Pu-erh, or Green Tea)], and S-adenosyl-L-methionine (SAMe)
*Indications*	*Dietary supplements for depression and anxiety*

### Adrenergic Pathways in the Modulation of the Immune Response

The two major pathway are involved in the brainimmune cross-talk are the hypothalamic-pituitary-adrenal axis and the sympathetic nervous system. The role of the sympathetic nervous system in the neuroimmune crosstalk has been the subject of several reviews (Elenkov et al., 2000; Nance and Sanders, 2007; Flierl et al., 2008; Cosentino and Marino, 2013; Marino and Cosentino, 2013). The predominant view includes the release of noradrenaline by sympathoadrenergic terminals, followed by activation of β_2_-ARs finally resulting into antiinflammatory effects (including to a variable extent the inhibition of T helper (Th) 1 proinflammatory cytokines such as IFN-γ, IL-12, TNF-α, and the enhancement of Th2 cytokines such as IL-10 and and transforming growth factor, TGF-β). Notably however noradrenaline may also promote IL-12-mediated differentiation of naive CD4+ T cells into Th1 effector cells which eventually produce IFN-γ (Swanson et al., 2001; Cosentino et al., 2013). Although β-ARs are considered the main interface between sympathoadrenergic terminals and immune cells, α-ARs may also occur in immune cells where they elicit proinflammatory responses, as in the case of α_1_-ARs on human macrophages (Grisanti et al., 2011) and of α_2_-ARs on rodent phagocytes (Flierl et al., 2007).

### Dopaminergic Pathways in the Modulation of the Immune Response

In comparison to noradrenaline and adrenaline, the immune effects of dopamine emerged only recently but very quickly attracted increasing attention (reviewed in Basu and Dasgupta, 2000; Sarkar et al., 2010; Levite, 2012). DR are expressed in most if not all human immune cells, including T and B cells, dendritic cells, macrophages, microglia, neutrophils and NK cells, and immune cells can “meet” dopamine not only in brain but also in blood, lymphoid organs and in several other peripheral tissues, such as the kidney and the hepatic vasculature (reviewed by Levite, 2012; Cosentino et al., 2013). Among human immune cells, CD4+CD25^high^ T lymphocytes are specifically sensitive to the activation of D_1_-like receptors expressed on their membrane, resulting in inhibition of the regulatory functions of this specialized cell subset, which usually suppresses the activity of effector T cells (Cosentino et al., 2007). Dopamine is also an emerging regulator of dendritic cell and T cell physiology, with critical implications for onset of immune-related disorders (Pacheco et al., 2009).

### Immune Cells as a Source of Dopamine, Noradrenaline and Adrenaline

**Figure 3:**
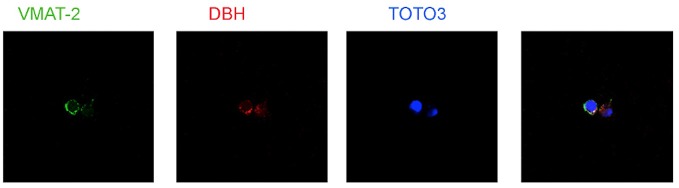
**Expression of vesicular monoamine transporters (VMAT2) and Dopamine β-hydroxylase (DBH) in human peripheral blood mononuclear cells (PBMCs)**. TOTO-3 iodide (642/660) was used for staining of nuclei. Cells were prepared at the Center for Research in Medical Pharmacology, University of Insubria, Varese (I) and analysis was performed at the Consorzio MIA—Microscopy and Image Analysis, Faculty of Medicine, University of Milan Bicocca, Milan (I).

Several types of immune cells may produce store and utilize catecholamines as autocrine/paracrine transmitters. The synthesis of dopamine, noradrenaline and adrenaline in immune cells likely occurs by means of a classical pathway, as suggested by the presence of the enzyme tyrosine hydroxylase (TH, EC 1.14.16.2), the first and rate-limiting enzyme in the synthesis of catecholamines, which undergoes upregulation following cell stimulation. TH inhibition, e.g., by α-methyl-*p*-tyrosine, prevents intracellular enhancement of catecholamines (Musso et al., 1996; Bergquist and Silberring, 1998; Cosentino et al., 1999, 2002a,b; Marino et al., 1999; Reguzzoni et al., 2002). In human peripheral blood mononuclear cells (PBMCs) stimulated *in vitro* with with phytohemagglutinin (PHA), TH mRNA expression and catecholamine production occur only in T and B lymphocytes (but not in monocytes) and are reduced by dopaminergic D_1_-like receptor activation (Ferrari et al., 2004), as well as by the proinflammatory cytokine IFN-γ, which in turn is counteracted by IFN-β (Cosentino et al., 2005). Human lymphocytes possess reserpine-sensitive compartments and vesicular monoamine transporters (VMAT) which are involved in intracellular storage of catecholamines (Marino et al., 1999; Cosentino et al., 2000, 2007; Figure 3). Catecholamine release can be induced by biological agents such as IFN-β (Cosentino et al., 2005) or by elevation of extracellular K^+^ ([K^+^]_e_; Cosentino et al., 2003). Human lymphocytes also express membrane transporter for dopamine (DAT; Marino et al., 1999; Marazziti et al., 2010) and for noradrenaline (NET; Audus and Gordon, 1982).

## Innervation of the BM and of other Hematopoietic Organs and Tissues

Primary lymphoid organs, such as BM and thymus, as well as secondary lymphoid organs, such as spleen and lymph nodes, are innervated by autonomic sympathoadrenergic efferent nerve fibers. The sympathetic nervous system and the hypothalamic-pituitary-adrenal axis are the major pathway connecting the CNS and the immune system (reviewed in Elenkov et al., 2000). Several excellent reviews discuss in detail the origin, distribution, signaling and targets of sympathetic nerves in lymphoid organs (Felten et al., 1985; Felten and Felten, 1988; Felten, 1991; Straub, 2004), the effect of age (Bellinger et al., 1992; Madden et al., 1995, 1997, 1998; Friedman and Irwin, 1997) and stress (Irwin, 1994; Marshall and Agarwal, 2000; Nagatomi et al., 2000; Sloan et al., 2008) as well as the relevance of dysregulated sympathetic nerovus system in immune-mediated disease (Bellinger et al., 1992, 2008; Madden et al., 1995; Friedman and Irwin, 1997; Marshall and Agarwal, 2000; Frohman et al., 2001; Straub et al., 2006; Wrona, 2006; del Rey and Besedovsky, 2008; Benarroch, 2009).

## Sympathoadrenergic Modulation of Hematopoiesis

Until the early 80 s, interest on adrenergic regulation of BM function was essentially concentrated on erythropoiesis (see e.g., Beckman et al., 1980; Lipski, 1980; Mladenovic and Adamson, 1984), with a few work dedicated to thrombocytopoiesis (Ganchev and Negrev, 1989).

Maestroni et al. (1992) were the first describing adrenergic modulation of hematopoiesis in an *in vivo* model, showing that chemical sympathectomy by 6-hydroxydopamine (6-OHDA) significantly increased the number of peripheral blood leukocytes after syngeneic BM transplantation in mice, an effect which was mimicked by the α_1_-AR antagonist prazosin. Results were reproduced in normal mice (Maestroni and Conti, 1994), by showing that prazosin can also enhance myelopoiesis and platelet formation, while noradrenaline and the α_1_-adrenergic agonist methoxamine could directly inhibit the *in vitro* growth of granulocyte/macrophage-colony-forming unit (GM-CFU). The order of potency of α-adrenergic antagonists on the effect of noradrenaline was prazosin>phentolamine>yohimbine. On these basis, the authors suggested that prazosin binds specifically to both BM cell membranes and intact BM cells, on two distinct binding sites, one with a K_d_ of 0.98 ± 0.32 nM and a B_max_ of 5 ± 2.9 fM/2 × 10^6^ cells (higher affinity site), and another with a K_d_ of 55.9 ± 8.2 nM and a B_max_ of 44 ± 7.7 fM/mg protein. Several lines of evidence suggest that the higher affinity site is actually an α_1_-AR, while the low affinity binding site remains to be characterized. The high-affinity binding is due to a lymphoid/stem cell fraction with no blasts and no GM-CFU progenitors, while the low-affinity site was apparent on a fraction enriched with GM-CFU progenitor cells (Maestroni and Conti, 1994). An initial summary of the significant evidence so far provided was published in Maestroni (1995), emphasizing the ability of α-AR antagonists to enhance myelopoiesis and platelets production while decreasing lymphopoiesis, in both normal mice as well as after BM transplantation. AR agonists, like the sympathetic neurotransmitter noradrenaline, seem to inhibit myelopoiesis, and effect which might be of clinical relevance, since it rescues the blood forming system and improves the survival of mice injected with a lethal dose of carboplatin or exposed to X-ray irradiation. This effect is apparently mediated by activation of α_1_-ARs expressed in pre-B cells, in turn inducing the production of TGF-β, which is finally responsible for the haematopoietic effects (Maestroni, 1995). Remarkably, it has been recently shown that nonmyelinating Schwann cells, which ensheath autonomic nerves in the BM, maintain HSC dormancy by activating latent TGF-β and that glial cell death and loss of HSC result from autonomic denervation of BM (Yamazaki et al., 2011). Noradrenaline was most effective at 3 mg/kg, s.c., and protected 77% of the mice injected i.v. with 200 mg/kg of carboplatin, which has a LD_100_ of 170 mg/kg. The effects was profoundly antagonized by the α_1_-AR antagonist prazosin. *In vitro*, 1 μM noradrenaline rescued GM-CFU in unseparated BM cells containing the adherent population expressing the high affinity α_1_-AR, another effect which was consistently counteracted by low concentrations of the α_1_-AR antagonist prazosin (0.1 nM-10 nM; Togni and Maestroni, 1996). Such results apparently challenge early reports suggesting that *in vitro* the β-AR agonist isoproterenol might result in increased proliferation and sensitivity of HSC to cytotoxic agents, an effect which was inhibited by the β-AR antagonist propranolol (Byron, 1972), however the studies cannot be directly compared due to fundamental differences in the experimental models and in the pharmacological agents employed. Interestingly, it was recently shown that also dopamine (50 mg/kg/days × 7 days i.p.), besides inhibiting tumor angiogenesis and growth of HT29 human colon cancer and Lewis lung carcinoma (LLC) in mice, also did not cause hypertension, hematological, renal and hepatic toxicities in normal, HT29 and LLC tumor bearing animals, and also prevented 5-fluorouracil (5FU) induced neutropenia in HT29 colon cancer bearing mice, an action apparently mediated through inhibition of 5FU mediated suppression of GM-CFU in the BM (Sarkar et al., 2014). In subsequent studies (Maestroni et al., 1997), it was further confirmed that noradrenaline administration in mice rescued hematopoiesis from the toxic effect of the chemotherapeutic agent carboplatin administered at supralethal doses (200 mg/kg), possibly by protecting GM-CFU. Meanwhile, Afan et al. (1997) reported that denervation decreases femoral cellularity as well as progenitor cells while mobilizing these cells in the peripheral blood of splenectomized mice. In non splenectomized animals, these changes were quickly cleared (Afan et al., 1997).

The consistent effects of noradrenaline and dopamine in the BM raised immediately the question regarding their physiological relevance and in particular the origin of catecholamines at this level. By use of a high performance liquid chromatographic method, we therefore measured endogenous catecholamines in BM from normal, 6-OHDA-treated and pargyline-treated mice. Noradrenaline levels were lower after 6-OHDA and higher after pargyline, while adrenaline and dopamine were not affected in either conditions (Marino et al., 1997). In the BM however noradrenaline, as well as the other catecholamines dopamine and adrenaline, may originate not only from nerve fibers but also from hematopoietic and immune cells themselves (Maestroni et al., 1998). In particular, in murine BM we described a daily rythmicity for noradrenaline and dopamine, with peak values occurring at night. Chemical sympathectomy disrupted the rhythm, whereas adrenaline showed no rhythmicity or 6-OHDA sensitivity. Noradrenaline was also positively associated with the proportion of cells in the G2/M and S phases of the cell cycle. Remarkably, in Méndez-Ferrer et al. (2008) published an elegant article suggesting just the opposite, i.e., that noradrenaline release in mouse BM is higher during the day/light hours. However, the findings of Maestroni et al. (1998) cannot be compared directly with those of Méndez-Ferrer et al. (2008) because the latter did not measure catecholamine concentration in the BM as Maestroni et al. did. The circadian release of noradrenaline was inferred by indirect experiments such as denervation, use of gene knock-out mice, and the catecholamine function was mimicked by injection of adrenergic agonists and/or antagonists. In addition, Maestroni et al. (1998) showed that BM cells themselves do contain catecholamines, therefore catecholamines in the BM resulted from both neural and hematopoietic cell contribution. Hence, Méndez-Ferrer et al. (2008) detected only one component of the system that was related to HSC trafficking while Maestroni et al. (1998) found a correlation between noradrenaline and BM cell proliferation. However, both groups found that chemical sympathectomy by 6-OHDA abolished the rhythm. Thus, a possible hypothetical interpretation that might reconcile these divergent findings is that the light/dark rhythm synchronizes the suprachiasmatic nucleus in the CNS which, in turn, entrains the sympathoadrenergic rhythm in the BM regulating the HSC traffic. In addition, the very same sympathetic nervous system or other circadian signals might affect clock genes in hematopoietic cell progenitors, influencing their noradrenaline content and their proliferation. Consistently, it has been reported that noradrenaline may affect clock genes expression (Morioka et al., 2010). Another circadian signal that ensues at the beginning of the activity period coinciding in rodents with the night is the adrenal corticosteroid output that is well known to affect clock genes expression. Interestingly, corticosteroids may also increase noradrenaline uptake in neuroblastoma cells (Sun et al., 2010) and this might happen also in BM cells containing catecholamines.

Circadian variation of the activity of sympathoadrenergic fibers innervating the bone may also affect bone homeostasis. Early studies indeed described increased bone remodeling during light periods in rodents (Simmons and Nichols, 1966). It is now established that β_2_-ARs are expressed in osteoblasts and osteoclasts and their stimulation triggers an osteoclastogenic response, while β_1_-AR actvation may result in bone protection, and even β_3_-ARs may indirectly affect skeleton homeostasis through their effects in other tissues (e.g., the adipose tissue). According to the current hypothesis, increased sympathetic activity could be associated with osteoporosis and the use of β-blockers might result in increased bone mineral density and decreased risk of fractures, although the clinical relevance of such effects is still under scrutiny (reviewed in Elefteriou et al., 2014).

Daily rythmicity of BM catecholamines likely contributes to the circadian control of the immune system, which is now emerging as important regulator of specific immune functions (Scheiermann et al., 2013). In addition, Maestroni et al. (1998) found noradrenaline and dopamine in both short-term and long-term BM cultures as well as in human or murine B lymphoid cell lines, an observation which subsequently prompted thorough investigation of endogenous production of catecholamines by immune cells (Marino et al., 1999). The ability of immune cells to produce and utilize catecholamines likely underlies novel opportunities for the targeted modulation of the immune response: as an example, we described in human CD4+CD25+ regulatory T lymphocytes the occurrence of an autocrine/paracrine loop involving dopaminergic pathways and resulting in down-regulation of their regulatory function (Cosentino et al., 2007), which is apparently involved in autoimmune disease such as multiple sclerosis (Cosentino et al., 2012).

In recent times, interest has risen for dopamine regulating bone marow hematopoiesis. By mans of flow cytometry Spiegel et al. (2007) showed that human CD34+ cells expressed both DR D_3_ and DR D_5_ on their surfaces. The more primitive CD34+CD38lo cell populations had higher expression of both DR D_3_ and DR D_5_ than did the more differentiated CD34+CD38hi cells. Interestingly, dopaminergic agonists increased the polarization and motility of CD34+ cells, as well as their clonogenic progenitor content and engraftment potential. In the same study, by means of flow cytometry, it was shown that human CD34+ cells expressed also the β_2_-AR, and G-CSF-mobilized CD34+ cells had higher expression of the β_2_-AR than did cord blood CD34+ cells. Adrenaline and noradrenaline regulated CD34+ cell motility and proliferation, *in vitro* as well as *in vivo*, possibily through a canonical Wnt signaling pathway (Spiegel et al., 2007).

## Effect of Stress on the Production of Inflammatory Cells

Recently, increasing attention has been dedicated to the mechanisms regulating the trafficking of HSC in the bloodstream. Giudice et al. (2010) reviewed the mechanisms regulating HSC trafficking, showing that circulating HSC exhibit marked circadian fluctuations due to standard cycles of 12 h light/12 h darkness and that circadian HSC oscillations are strongly altered when mice are subjected to continuous light for 2 weeks or to a jet lag. HSC fluctuation is likely in antiphase with the expression of the chemokine CXCL12 in the BM microenvironment. Both circadian HSC trafficking and expression of CXCL12 are modulated by rhythmic release of sympathoadrenergic transmitters in the BM (Giudice et al., 2010). Several lines of evidence indeed suggest that hematopoiesis may be subject to catecholaminergic regulation even under extreme conditions, such as restraint stress and cytostatic treatment (Dygai and Skurikhin, 2011), although also the stress hormone corticosterone may exert major effects on HSC in the BM, as suggested by increased HSC apoptosis and reduced BM repopulation and stromal progenitor cell number following high corticosterone exposure and, on the other side, increased BM HSC and CXCL12 levels in animals with low corticosterone levels or treated with the corticosterone synthesis inhibitor metyrapone (Kollet et al., 2013). Indeed, transcriptome representation analyses showed relative expansion of the selective up-regulation of a subpopulation of immature proinflammatory monocytes (Ly-6c(high) in mice, CD16(-) in humans) within the circulating leukocyte pool in peripheral blood mononuclear cells from people subject to chronic social stress (low socioeconomic status) and mice subject to repeated social defeat (Powell et al., 2013). The effect was ascribed to increased myelopoietic output of Ly-6c(high) monocytes and Ly-6c(intermediate) granulocytes in mice subject to repeated social defeat, and was blocked by treatment with β-AR antagonists as well as with the myelopoietic growth factor GM-CSF. On these basis the authors suggest that sympathoadrenergic-induced up-regulation of myelopoiesis results in a proinflammatory response possibly contributing to the increased risk of inflammation-related disease associated with adverse social conditions (Powell et al., 2013). The ability of chronic stress to induce monocytosis and neutrophilia in humans has been recently reproduced comparing medical ICU residents either off duty or on ICU duty (Heidt et al., 2014), and by use of rodent models it was shown that under conditions of chronic variable stress in mice, sympathetic nerve fibers increase the release of noradrenaline, which in turn signals BM niche cells to decrease CXCL12 levels through β_3_-ARs. This leads to increased HSC proliferation and subsequently increased output of neutrophils and inflammatory monocytes (Heidt et al., 2014). Interestingly, treatment of mice with noradrenaline, mimicking acute stress, has been reported to increase circulating levels of CXCL12, resulting in rapid mobilization of HSC, an effect which is induced also by plerixafor (AMD3100), an immunostimulant used to mobilize HSC, and blocked by injection of a β_2_-AR antagonist (Dar et al., 2011), suggesting that acute and chronic stress may result in different effects on the BM. The pathological implications of chronic stress-induced monocytosis and neutrophilia were tested in atherosclerosis-prone Apoe(–/–) mice which, when subjected to chronic stress, accelerated hematopoiesis and promoted plaque features associated with vulnerable lesions that cause myocardial infarction and stroke in humans (Heidt et al., 2014). Interestingly, a similar mechanism is likely involved in the expanded neutrophil and monocyte supply which may occur after stroke (Courties et al., 2015). Indeed, in mice with transient middle cerebral artery occlusion (tMCAO), flow cytometry and cell cycle analysis showed activation of the entire hematopoietic tree, including myeloid progenitors resulting in increased expression of myeloid transcription factors, including PU.1, and declined lymphocyte precursors. Notably, In mice after tMCAO, the levels of TH (the first and rate-limiting enzyme in the synthesis of catecholamines) rose in sympathetic fibers and BM noradrenaline levels increased, ass hematopoietic niche factors that promote stem cell quiescence decreased. In mice with genetic deficiency of the β_3_-AR, on the contrary, HSCs did not enter the cell cycle in increased numbers after tMCAO (Courties et al., 2015).

## Repurposing of Adrenergic and Dopaminergic Agents as Modulators of Hematopoiesis in Health and Disease

The possibility to manipulate hematopoiesis by means of sympathoadrenergic mechanisms provides enormous therapeutic opportunities, also in view of the great amount of adrenergic and dopaminergic agoniststs and antagonistts and indirectly acting agens which are altready in clinical use with a usually favorable therapeutic index (Marino and Cosentino, 2013). Recently, Lucas et al. (2013) provided further cofirmation that that sympathoadrenergic innervation of the BM is crucial for hematopoietic regeneration after chemotherapy. Maestroni et al. (1992) however, who first described *in vivo* the adrenergic modulation of hematopoiesis, showed that chemical sympathectomy by 6-hydroxydopamine (6-OHDA) increased peripheral blood leukocytes after syngeneic BM transplantation in mice. Lucas et al. (2013), on the contrary, reported reduced survival in 6-OHDA-treated animals, and differences are hardly explained by experimental conditions, as both mice strain and gender, as well as chemical denervation protocol, are the same (except for additional 250 mg/kg of 6-OHDA on day 2 after the initial 100 mg/kg on day 0). In addition, in the study by Maestroni et al. (1992) differences between saline- ad 6-OHDA-treated animals were evident only in animals kept under continuous 24-h lighting, and not in those kept under a 12:12 light:dark cycle. Finally, in the study by Maestroni et al. (1992) the effect of sympathetic denervation was concentration-dependently mimicked by the α_1_-AR antagonist prazosin, while the non selective β-AR antagonist propranolol was without effect *per se* and selectively reverted the effect of prazosin on platelets. Lucas et al. (2013) used only β_2_- and β_3_-AR antagonists, and only at one dose, which resulted in mild effects qualitatively similar to those of 6-OHDA. It is possible that differences in the effects of 6-OHDA on BM and circulating cell recovery may depend upon the different doses used. In the article by Lucas et al. (2013) we found no information concerning the actual effects of 6-OHDA on TH+ nerve endings in BM or on blood cells. Indeed, 6-OHDA can exert direct toxicity on circulating lymphocytes (Del Rio and Velez-Pardo, 2002), and high doses might be therefore less selective for nerve endings. Anyway, clarifying such minor methodological and procedural issues will pave the way for clinical trial of adrenergic agents as promoters of hematopoietic recovery.

Evidence obtained in rodents indicate that β_2_-AR agonists may enhance mobilization of HSC and hematopoietic progenitor cells. Katayama et al. (2006) showed that after administration of the β_2_-AR agonist clenbuterol, the mobilization defect was partly rescued in *Dbh*^−/−^ mice (lacking dopamine β-hydroxylase, the enzyme which converts dopamine into noradrenaline) and resulted in enhanced mobilization in *Dbh*^+/−^ animals. Clenbuterol was effective only before and during G-CSF administration. The authors propose that the effect of G-CSF is due to release of noradrenaline from sympathetic nerve endings resulting in osteoblast suppression and reduced synthesis of CXCL12, through the activation of β_2_-ARs which cooperate with other signals from the G-CSF receptor (Katayama et al., 2006).

Sympathoadrenergic agents may also contribute to restore normal hematopoiesisis in myeloproliferative neoplasms. Sympathoadrenergic fibers, supporting Schwann cells and nestin(+) mesenchymal stem cells are reduced in the BM of patients with myeloproliferative neoplasms as well as in mice expressing the human JAK2(V617F) mutation in HSCs. Interestingly, reduction of mesenchymal stem cells is due to BM neural damage and Schwann cell death triggered by IL-1β, resulting in expanded mutant HSC number and accelerated myeloproliferative neoplasms progression. Treatment with β_3_-AR agonists restore the sympathetic regulation of nestin(+) mesenchymal stem cells, blocking myeloproliferative neoplasms progression by indirectly reducing the number of leukaemic stem cells (Arranz et al., 2014).

Neuropathy of sympathoadrenergic fibers has been recently proposed also as a novel mechanism for malignancies like acute myelogenous leukemia (AML) to exploit the hematopoietic microenvironment for its purposes (Hanoun et al., 2014). Indeed, in an animal model of AML, of sympathetic nervous system neuropathy promotes leukemic BM infiltration, possibly through an expansion of perivascular mesenchymal stem and progenitor cells primed for osteoblastic differentiation at the expense of the physiological periarteriolar niche cells. Blockade of β_2_-AR pathways enhanced AML infiltration whereas a β_2_-AR agonist reduced disease activity.

As a final remark, we would like to mention the recently emerging evidence which indicate the multiple ways in which the local microenvironment may contribute to cancer-induced bone disease, possibly through a key role of the sympathetic nervous system providing bone homeostatic signals. Stress and anxiety are able to cause bone loss through the sympathetic nervous system, and have been shown to have an effect on not only the osteolytic effect of breast cancer, but also the metastatic infiltration of bone. Sympathetic nervous system signaling to β-ARs on osteoblasts has also been implicated in potentiating other signals, such as parathyroid hormone, osteopontin and IGF-1, and release of HSCs from their niche, which may also have implications for invading cancers. Preliminary evidence have been recently summarized into an excellent review (Olechnowicz and Edwards, 2014) and the topic will likely attract the broadest interest in the near future.

## Conclusion

Although the first *in vivo* evidence for the role of sympathoadrenergic fibers in the modulation of hematopoiesis was provided less than 25 years ago (Maestroni et al., 1992), the relevance of catecholaminergic modulation of hematopoiesis rapidly raised thanks to several seminal studies showing the key role of sympathoadrenergic fibers in the hematopoietic niche, as well as the potential of adreneceptor ligands, and in some cases even of dopamine receptor ligands (Sarkar et al., 2014).

In addition, the recently established notion that activation of sympathoadrenergic represents a link between chronic stress (e.g., due to adverse social conditions) and up-regulation of proinflammatory responses, such as monocytosis and neutrophilia in humans (see e.g., Heidt et al., 2014), not only provides a mechanistic explanation to the negative prognostic role of the neutrophil/lymphocyte ratio in a broad and heterogeneous number of critical conditions, such as cancer (Templeton et al., 2014) and cardiovascular disease (Guasti et al., 2011; Bhat et al., 2013) but also offers several opportunities for therapeutic intervention. Results obtained so far in preclinical models would already support to various extent the clinical evaluation of: the α_1_-AR antagonist prazosin (Maestroni et al., 1992; Maestroni and Conti, 1994), β_2_-AR agonists (Katayama et al., 2006; Dar et al., 2011) and dopaminergic agonists (Spiegel et al., 2007) for HSC transplantation; α_1_-AR agonists (Togni and Maestroni, 1996; Maestroni et al., 1997) as well as dopaminergic agonists (Sarkar et al., 2014) to protect against the adverse effects of cytotoxic agents on BM; β-AR antagonists to reduce the proinflammatory response associated with chronic stress (Powell et al., 2013; Heidt et al., 2014); β_2_-AR agonists (Hanoun et al., 2014) and β_3_-AR agonists (Arranz et al., 2014) in myeloproliferative disease.

Sympathoadrenergic innervation has finally reached an established role in the complex network of neural and neuroendocrine agents which regulate the hematopoietic system (Maestroni, 2000). Several key questions still await answers, including whether the neural regulation of hematopoiesis plays any role in aplastic anemia, leukemia, and immune-based diseases or during emergencies such as acute infections and/or stress events: any positive response will provide the conceptual framework for the straightforward development of new pharmacological strategies, considering the availability of several dopaminergic and adrenergic agents, already in clinical use for non-immune indications and with a usually favorable risk-benefit profile. Finally, from a general point of view, these findings include hematology among the fields which cannot but benefit from an integrative neuroimmune pharmacological approach (Izeku and Gendelman, 2008).

## Conflict of Interest Statement

The authors declare that the research was conducted in the absence of any commercial or financial relationships that could be construed as a potential conflict of interest.
